# Statins in Neurological Disorders: Mechanisms and Therapeutic Value

**DOI:** 10.1100/tsw.2009.141

**Published:** 2009-11-01

**Authors:** Allison B. Reiss, Elzbieta Wirkowski

**Affiliations:** ^1^ Vascular Biology Institute, Department of Medicine, Winthrop-University Hospital, Mineola, New York, USA; ^2^ Division of Neurology, Department of Medicine, Winthrop-University Hospital, Mineola, New York, USA

**Keywords:** statins, stroke, Alzheimer’s disease, cholesterol, amyloid beta, inflammation

## Abstract

Statins are well-tolerated, mainstay drugs in cardiovascular risk management. In addition to their cholesterol-lowering properties, statins also have anti-inflammatory, vasculoprotective, and antioxidant effects. They have also been associated in some epidemiologic studies with reduced risk of Alzheimer's disease (AD), and a link between cholesterol and late-onset AD has been documented. Experimental studies in cell culture systems and animal models show that statins have neuroprotective effects that may ameliorate the damage inflicted by stroke and AD. Human studies have garnered compelling evidence that treatment with statins reduces ischemic stroke incidence independent of their lipid-lowering effects. There is also the possibility that statins and extremely low cholesterol levels may increase the risk of intracranial hemorrhage. In this review, we discuss the potential reasons for the effect of statins on stroke and AD, and the multiple mechanisms of action of this class of lipid-lowering drugs.

## INTRODUCTION

Statins, or 3-hydroxy-3-methylglutaryl-coenzyme A (HMG-CoA) reductase inhibitors, are widely prescribed as cholesterol-lowering therapy, and are considered first-line therapeutic agents for the prevention of coronary heart disease and atherosclerotic disorders related to hypercholesterolemia[1]. They are administered orally and have dose-dependent benefits in reducing plasma concentrations of cholesterol[2]. They are used for hyperlipidemia and for the primary and secondary prevention of cardiovascular (CV) disease, and clinical trials have substantiated their effectiveness in lowering mortality levels associated with CV disease[3,4].

Statins also have immunomodulatory, neuroprotective, and anti-inflammatory properties that are being explored for potential benefits in central nervous system disorders[5,6,7]. The etiology of a number of neurologic disorders involves both vascular dysfunction and immunopathology, and this has led to the use of statin therapy, with varying degrees of success, in neuroinflammatory and neurodegenerative processes. Statins may also be used to reduce mortality and neurological disability from stroke, and to reduce the incidence of dementia, although the latter is controversial[8,9].

This review summarizes the data concerning statin use in the prevention and treatment of neurological disorders. Effective treatment of these disorders, including stroke, Alzheimer's disease (AD), Parkinson’s disease, and multiple sclerosis, remains a challenging task in clinical practice.

## PHARMACOLOGICAL PROPERTIES

The therapeutic target organ of statins is the liver, where the drugs act on hepatocytes to inhibit endogenous cholesterol biosynthesis. This blockade results in decreased intracellular levels of cholesterol in the hepatocytes, which leads to activation of the sterol regulatory element binding protein (SREBP) pathway and compensatory up-regulation of low-density lipoprotein (LDL) receptors on the cell surface. Consequently, hepatic uptake and catabolism of LDL increases[10,11].

Six different statins (simvastatin, pravastatin, lovastatin, fluvastatin, rosuvastatin, and atorvastatin) are approved for treatment of hypercholesterolemia in humans. These statins share an HMG-like moiety and act as competitive inhibitors of HMG-CoA reductase, an enzyme of the endoplasmic reticulum that catalyzes conversion of HMG-CoA into mevalonic acid, the rate-limiting step in cholesterol biosynthesis[12] (Fig. 1). Statins bind to HMG-CoA reductase at nanomolar concentrations, leading to competitive displacement of the natural substrate, HMG-CoA, which binds at micromolar concentrations[12]. They thus prevent substrate access to active sites of the enzyme, and this results in a reduction of serum total and LDL cholesterol. Intermediate statin doses lower LDL by about 30–40%, while high dose therapy lowers LDL by about 50%[13,14].

Statins are categorized based on criteria such as their origin, their hydrophilicity/hyrophobicity, and their specificity. Lovastatin, pravastatin, and simvastatin are all obtained by fungal fermentation, while atorvastatin, fluvastatin, rosuvastatin, and cerivastatin (withdrawn from the market in 2001) are entirely synthetic[15,16]. All statins, both fungal metabolites and synthetic compounds, confer similar coronary heart disease event reduction when adjusted for differences in lipid changes[17]. Pravastatin and rosuvastatin are hydrophilic; fluvastatin has intermediate characteristics; lovastatin, simvastatin, atorvastatin, and cerivastatin are hydrophobic[16,18]. Atorvastatin, cerivastatin, fluvastatin, rosuvastatin, and pravastatin are administered as orally active beta-hydroxy acid compounds (acid form). Lovastatin and simvastatin are administered as inactive prodrugs (lactone), which have to be enzymatically hydrolyzed to generate active forms[19]. Atorvastatin, lovastatin, simvastatin, and cerivastatin are metabolized by the cytochrome P-450 isozyme 3A4. Unlike these lipophilic statins, hydrophilic statins (such as pravastatin) are not metabolized in the liver via the cytochrome P-450 system and have little potential for adverse events through interaction with drugs metabolized via this pathway[16].

## CHOLESTEROL-INDEPENDENT STATIN EFFECTS

Statins exhibit multiple nonlipid-lowering actions or “pleiotropic” effects (Table 1)[20]. These beneficial effects that extend beyond their cholesterol-lowering capacity include, among others, vasodilation, plaque stabilization, inhibition of thrombogenesis, attenuation of oxidative stress, and reduction of inflammation[21,22,23]. The anti-inflammatory effects include suppression of the release of proinflammatory cytokines, chemokines, adhesion molecules, and matrix metalloproteinases (MMPs) by inflammatory cells[24,25]. Statins can inhibit MMP-2 and MMP-9 activities by monocytes, macrophages, fibroblasts, and vascular smooth muscle cells[25]. Statins act as immunomodulators by suppressing T-cell activation and proliferation, and by reducing the expression of class II major histocompatibility complexes on antigen-presenting cells[26,27]. Antioxidant actions lead to reduced superoxide production and lipid peroxidation[28,29]. Simvastatin selectively blocks the production of free oxygen radicals generated by the stimulated macrophages[30,31].

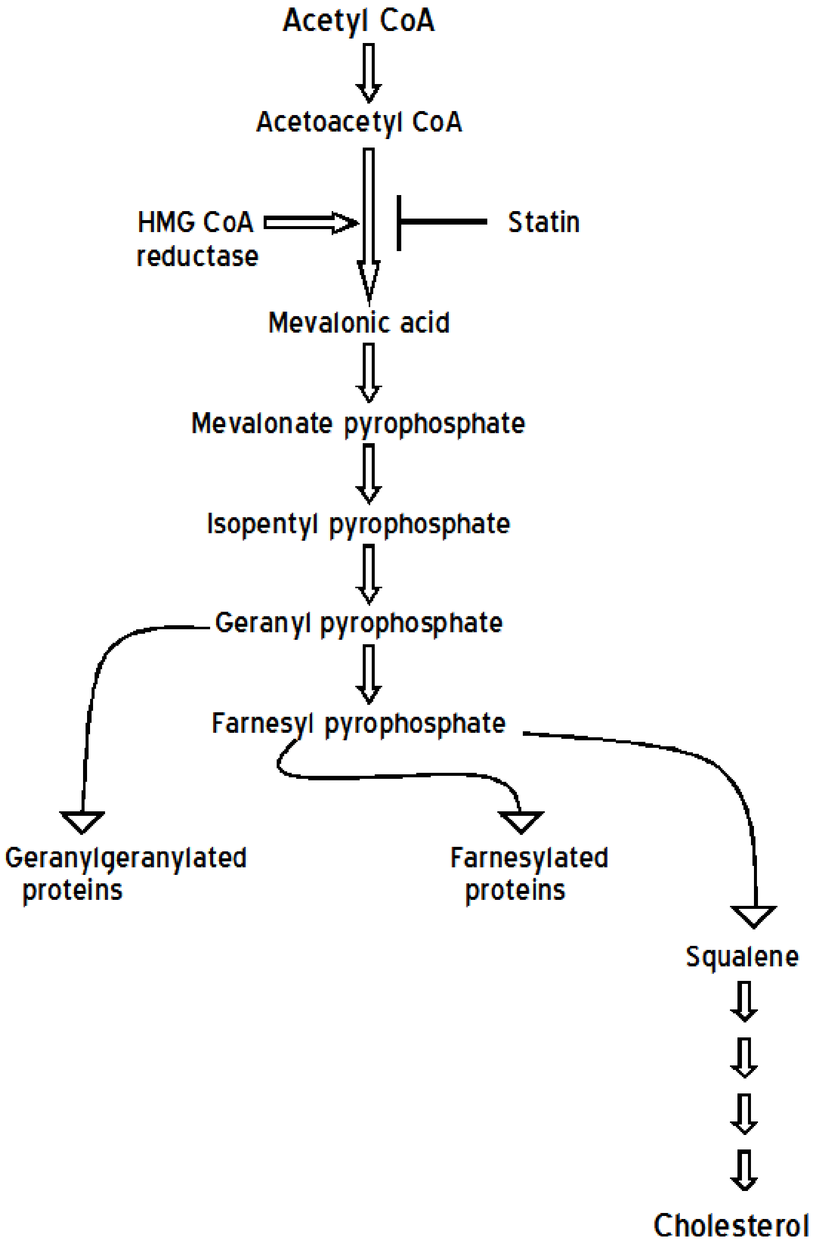

**FIGURE 1.** Cholesterol biosynthetic pathway. All carbon atoms in cholesterol are derived from acetyl CoA. The synthesis of mevalonic acid is catalyzed by the enzyme HMG-CoA reductase. It is the committed, rate-limiting step in cholesterol formation. This enzyme is highly regulated and the target of pharmaceutical intervention with statins. Mevalonic acid is converted to mevalonate pyrophosphate and then isopentyl pyrophosphate via ATP-requiring reactions. Six isopentyl pyrophosphates are isomerized and condensed to form squalene. The 30-carbon linear squalene undergoes cyclization to form the four rings of the steroid nucleus, with a further series of changes to yield the 27-carbon cholesterol molecule. Alternatively, through a series of other intermediates, mevalonic acid can be converted to geranyl pyrophosphate and farnesyl pyrophosphate, metabolic products that are able to supply prenyl groups for protein prenylation. Prenylated proteins have covalently linked geranylgeranyl or farnesyl groups that anchor those proteins to membranes. Prenylation is a post-translational lipid modification that affects the function of Ras, Rho, and other small GTPases.

**TABLE 1**
**Pleiotropic Effects of Statins with Neuroprotective Benefits**

| |
|---|
| Increasing NO bioavailability, which augments cerebral perfusion |
| Inhibition of isoprenylation of RhoA GTPase, which increases production of NO by eNOS |
| Modulation of inflammatory response with decreased production of proinflammatory cytokines |
| Inhibition of NF-kB activation, which influences inflammatory gene transcription |
| Blocking cell-surface expression of adhesion molecules |
| Decreasing susceptibility to thrombosis |
| Decreasing platelet aggregation |
| Reducing thrombin formation |
| Enhancing clot lysis |
| Stabilization of atherosclerotic plaques |
| Antioxidant effects with decreased ROS |

## The Isoprenoids and Pleiotropic Effects

Many of the so-called pleiotropic effects have been shown to be secondary to the inhibition of the synthesis of isoprenoid intermediates of the mevalonate pathway (Fig. 1). This important class of biomolecules, such as farnesyl pyrophosphate (FPP) and geranylgeranyl pyrophosphate (GGPP), are essential for the post-translational modification of small guanosine triphosphate (GTP)–binding proteins, such as Ras, Rac, and Rho. Ras small G-proteins are modified by either farnesylation or geranylgeranylation, whereas Rho small G-proteins are modified by geranylgeranylation. Addition of these isoprenoids to the C termini of small G-proteins is critical for enabling translocation and tethering of these proteins to the cell membrane, and allowing their subsequent interaction with effector molecules and cytoplasmic regulators through protein-protein and protein-lipid interactions[32,33,34]. Rho (together with its downstream target, Rho-associated coiled-coil protein kinase[ROCK]) and Rac regulate organization of cytoskeletal proteins involved in endothelial cell barrier function and are involved in inflammatory signal transduction pathways[35]. Rho participates in signaling pathways required for nuclear factor-kappa B (NF-kB) activation, leading to the induction of cytokines and chemokines[36]. Statins deplete cells of all downstream isoprenoid products, causing guanosine triphosphatase (GTPase) proteins to lose their normal membrane association and function, leading to cytosolic accumulation of these small GTPases[37,38]. In cultured human pulmonary artery endothelial cells, simvastatin inhibited the geranylgeranylation of Rac1, associated with a paradoxical increase in Rac1 GTP binding and activation[39]. The activation of Rac1 is a key step in activation of endothelial nitric oxide synthase (eNOS), responsible for production of neuroprotective nitric oxide (NO). Silencing (si)RNA–mediated Rac1 “knockdown” in endothelial cells markedly suppresses receptor signaling to eNOS[40].

Statins increase the production of vasodilators, such as NO, prostaglandin $I_2$, and vascular endothelial growth factor, and decrease the production of vasoconstrictors, such as endothelin-1 and angiotensin II, the terminal pressor effector hormone of the renin-angiotensin-aldosterone system. The angiotensin II octapeptide increases blood pressure by vasoconstriction and sodium and fluid retention, and acts in a pro-oxidative capacity by generating reactive oxygen species (ROS)[41]. Statins attenuate the deleterious effects of the renin-angiotensin system in several organs, not only by decreasing angiotensin II, but by down-regulating expression of the angiotensin II type 1 (AT1) receptor[42,43]. In vascular smooth muscle cells isolated from the thoracic aorta of Sprague-Dawley rats, cerivastatin reduced AT1 receptor promoter activity and decreased AT1 receptor expression in a dose-dependent fashion, and the addition of GGPP, but not FPP, was able to prevent cerivastatin-induced down-regulation of AT1 receptor expression[44].

### Statins and Nitric Oxide

Normal endothelial function is required to maintain vascular homeostasis and is dependent on NO. NO, generated from L-arginine through the actions of the enzyme NO synthase (NOS), modulates ROS production, blood flow, platelet function, vascular permeability, and vascular tone[45,46]. NO limits inflammation and coagulation, and diminishes vascular smooth muscle cell proliferation and migration[47,48]. Statins are known to rapidly increase NO bioavailability through multiple mechanisms. This is one of the earliest effects of statin treatment[49]. NO is synthesized by three different NOS isoforms: eNOS, inducible NOS (iNOS), and neuronal NOS (nNOS).

Statins enhance iNOS expression in vascular smooth muscle cells and eNOS in endothelial cells by cholesterol-independent mechanisms involving Rho and requiring geranylgeranylation, leading to prolongation of the half-life of the NOS mRNA[50,51,52]. Statins can also rapidly induce the phosphorylation and activation of eNOS via the phosphatidylinositol-3 kinase (PI3K)/protein kinase Akt pathway. Rodents treated with statins display elevated endothelial and platelet eNOS levels, enhanced NO production, and protection against cerebral ischemia[53,54,55]. In rat vascular smooth muscle cells, atorvastatin increases expression of both iNOS and nNOS mRNA and protein via the Akt/NF-kB pathway[56]. Corroborating the rodent and cell culture studies, statin effects on platelet and endothelial eNOS have also been observed in humans[57,58]. Pravastatin treatment potentiates human platelet eNOS activity in humans[59].

Many of the pleiotropic effects of statins are neuroprotective, such as the ability to scavenge ROS and MMPs, reduce thrombogeniciity, facilitate clot lysis, up-regulate eNOS, and modulate the inflammatory response[60,61,62].

## IMPACT OF STATINS ON ALZHEIMER'S DISEASE

AD, the leading cause of dementia worldwide, is a chronic progressive condition characterized by cognitive decline and impaired memory, thinking, and behavior[63]. Morphological alterations considered as hallmarks of AD include amyloid plaques and neurofibrillary tangles (intracellular filamentous aggregates of the microtubule-associated protein tau), accompanied by neuroinflammation, neuronal dysfunction, and cell death. These plaques and tangles constitute potential targets for AD therapy. The 40–42 amino acid peptide, called beta-amyloid (Abeta), has been identified as a main component of amyloid plaques and is centrally implicated in the pathogenesis of AD[64]. Proteolytic processing of the amyloid precursor protein (APP) by the sequential activities of beta- and gamma-secretase releases the potentially neurotoxic Abeta peptide, which is present in the brain in both its soluble and aggregated (senile plaques) forms[65]. APP can also be processed through the alpha-secretase pathway in which APP is cleaved in the middle of the Abeta region, thus precluding formation of Abeta[66].

### The Link Between Apolipoprotein (Apo) E and Alzheimer's Disease

ApoE is a major apolipoprotein in the brain and is involved in lipid transport and neuronal homeostasis[67]. ApoE participates in the transport of cholesterol and other lipids in the bloodstream and in the cerebrospinal fluid (CSF), and is the most common genetic risk factor for late-onset sporadic AD[68]. This protein is expressed by three major alleles and exists in three isoforms, apoE2, apoE3, and apoE4, which vary by single amino acid changes in their structure. Of the three forms, only the E4 allele increases the likelihood of developing AD and is the strongest known genetic risk factor for development of late-onset AD[69,70]. Results from the Alzheimer's Disease Cholesterol-Lowering Treatment (ADCLT) trial suggest that statin therapy may be of particular benefit for patients with mild-to-moderate AD with the ApoE4 allele who have elevated serum cholesterol[71]. The Prospective Study of Pravastatin in the Elderly at Risk (PROSPER) found no such benefit[72,73]. PROSPER confirmed the association between the apoE4 allele and decline in global cognitive function, even in subjects not diagnosed with dementia. This randomized controlled trial followed a large cohort (5804 subjects aged 70–82, 50% female) over a brief period (average 3.2 years). Half of the subjects received pravastatin, but the drug did not slow cognitive decline in any subgroup, including those with the apoE4 allele, nor did lipoprotein levels influence cognitive decline. The study did not address the effect of statins in early or mid-life age groups.

## Cholesterol Metabolism and Alzheimer's Disease

The role of cholesterol metabolism in the etiopathogenesis of AD is of intense interest and is being explored *in vitro*, and in humans and pertinent animal models. Neurobiochemical studies have shown that the lipid environment modulates APP processing[74]. Membrane cholesterol promotes amyloid production through processing of the type I transmembrane protein APP by the integral membrane proteins beta- and gamma-secretase, and can therefore increase generation of toxic beta-amyloidogenic peptides. Beta-secretase activity is thought to occur primarily in lipid rafts, which are detergent-insoluble microdomains rich in cholesterol and sphingomyelin[75]. Reduction in cholesterol concentration decreases beta-secretase activity[76]. Depletion of membrane cholesterol from buoyant membrane microdomains derived from cultured cells with methyl-beta-cyclodextrin completely inhibits gamma-secretase cleavage activity, which can then be restored by cholesterol replenishment[77]. The effects of cholesterol depletion on these secretases are independent of each other[78]. Upon membrane cholesterol depletion by either pharmacological or biochemical means, alpha-secretase activity is enhanced and Abeta production diminished[79,80]. Thus, cholesterol levels may influence the balance between amyloidogenic and amyloidolytic (nonamyloidogenic) proteolytic processing of APP[75].

In cells in culture, reducing cholesterol levels with statins causes a marked reduction in Abeta[76,81]. Statins can affect both beta- and gamma-secretase activities, thereby decreasing the breakdown of APP, and it has been proposed that this may lead to reduction in the risk of AD[76,82]. Amyloidogenesis under conditions of cholesterol reduction may also be averted due to statin-induced up-regulation of alpha-secretase[80,83]. Gellermann and colleagues[84] found that lovastatin reduced the formation of amyloid-like Abeta plaques by cultured primary human macrophages by 35%. Microglia mount a robust phagocytic response to Abeta, but when exposed to statins, they exhibit reduced phagocytosis with Abeta stimulation and this effect occurs via depletion of isoprenoid precursors[85].

In murine studies, diet-induced hypercholesterolemia increases Abeta accumulation and accelerates AD-related pathology[86,87]. Similarly, in New Zealand White rabbits, a cholesterol-enriched diet increased intraneuronal Abeta immunoreactivity. Upon removal of cholesterol from the diet of these animals, a reduction in brain Abeta levels was observed along with activation of microglia[88].

Several murine studies show a salutary effect of statins on amyloid deposition[81,89]. Other studies have shown no benefit or added amyloid burden[90,91]. In transgenic mice overexpressing human APP, lovastatin or pravastatin treatment both induced dose-dependent reductions in total Abeta peptides[92]. In a transgenic mouse model of taupathy, atorvastatin and simvastatin were both effective in reducing neurofibrillary tangle burden[93].

Li and colleagues[90] evaluated female mice that overexpress the human APP with a Swedish double mutation (Tg2576 mice) for behavior and cerebral amyloidosis. They compared Tg2576 mice treated with 50 mg simvastatin per kilogram body weight per day for 3 months to mice not receiving the drug, and found reversal of spatial learning and memory deficits in treated mice. Despite improved cognitive function, the simvastatin-treated Tg2576 mice failed to show a significant decrease in brain Abeta level. When male and female Tg2576 mice were given lovastatin (100 mg/kg/day for 3 weeks), Park et al.[91] found that the response differed by gender with increased Abeta production and plaque deposition in female, but not in male mice. The overall effect of statins on Abeta in mice has not been resolved.

The association between cholesterol and cognition is complicated and the efficacy of statins at slowing the progression of AD remains controversial. A number of epidemiological studies have demonstrated that chronically elevated serum total cholesterol is a risk factor for developing AD later in life[94,95,96]. Launer and colleagues found that low mid-life total cholesterol was associated with fewer numbers of dendritic plaques and tangles[97]. In a postmortem study of 100 individuals, Kuo et al. observed significantly higher LDL and significantly lower HDL in the documented AD cases vs. controls[98]. In contrast, a number of other studies, including the Framingham study, have failed to detect an association between raised total cholesterol and AD[99,100]. Overall, whether hypercholesterolemia is a clinical risk factor for AD is still unresolved.

## Statins in Alzheimer's Disease: The Human Studies

Past findings with respect to reduction in AD risk with statin use have been inconsistent[101]. However, several recent prospective studies have reported that statin use lowers AD incidence. Results from the Sacramento Area Latino Study on Aging (SALSA), a prospective cohort observational study of older (≥60 years of age) Mexican Americans from the Sacramento, CA area, indicate that in this population, statin use was associated with a significant reduction in the incidence of combined dementia and cognitive impairment without dementia (CIND). Over a 5-year period, rates of dementia/CIND were 44% lower in statin users[9]. In the Rotterdam Study, a large clinical prospective study, Haag and colleagues[102] found that over an average 9.2 years of follow-up of persons with an average age of 69.4 at baseline, statin treatment reduced the risk of incident AD regardless of apoE genotype, and the protective effect of statins was shown to be independent of their lipophilicity. Nonstatin cholesterol-lowering drugs (fibrates, bile-acid binding resins, or nicotinic acid and derivatives) failed to provide protection. In the Alzheimer's Disease Anti-inflammatory Prevention Trial (ADAPT) study, a prospective observational study of elective statin use in an at-risk-for-AD population of elderly individuals, statin use was associated with significantly reduced risk of incident AD after adjustment for confounding variables[103].

Any protective actions of statins may be a direct consequence of reducing cholesterol levels and may also be independent of cholesterol lowering[104]. Small doses of statins below the threshold for impacting cholesterol levels can block isoprenoid synthesis in neuroblastoma cells, interfering with APP trafficking and inhibiting amyloidogenic APP processing[105]. In cultured hippocampal neurons, lovastatin inhibited APP endocytosis in a GGPP-dependent manner, causing decreased generation of Abeta[106]. Addition of GGPP to APP overexpressing cells treated with lovastatin fully reverses the lovastatin-mediated reduction in Abeta[107]. On the whole, accumulated evidence in cell culture experiments indicates that statins attenuate the amyloidogenic process by inhibiting both cholesterol and isoprenoid biosynthesis[108,109].

Despite some biochemical rationale for statin effectiveness in AD and the positive results obtained in SALSA, ADAPT, and the Rotterdam Study, conflicting data continue to be revealed. Two double-blind, randomized, placebo-controlled trials of statins (PROSPER, which was previously mentioned, and the Heart Protection Study [HPS]) were analyzed and neither showed any impact of statins on cognitive function[73,110]. PROSPER included 5804 patients ranging in age from 70 to 82 years with mean follow-up of 3.2 years, and the HPS was comprised of 20,536 patients with 5806 patients age 70 years or above at study entry and mean follow-up of 5 years. PROSPER patients received pravastatin or placebo, while HPS patients received simvastatin or placebo. In the Religious Orders Study, a prospective clinical-pathologic study of dementia in older (74.9 year average age at baseline) Catholic clergy from across the U.S. without signs of dementia at baseline, no relationship was found between statin use and incidence of AD[111]. Despite a 12-year follow-up, no effect of statins was found on AD or cognition, and no relation of statins to tangles or amyloid load.

Results are also starting to emerge from both the Lipitor's Effect in Alzheimer's Dementia (LEADe) Study and the Cholesterol Lowering Agent to Slow Progression of AD (CLASP) study[112,113]. LEADe is an 80-week, international, multicenter, randomized, double-blind, placebo-controlled study of atorvastatin 80 mg daily vs. placebo in patients age 50–90 years (mean age 74 years) with mild-to-moderate AD receiving background therapy of donepezil 10 mg daily. In total, 641 subjects were enrolled and, thus far, there has been a lack of significance of the positive signal of benefit on the Alzheimer's Disease Assessment Scale-cognitive subscale (ADAS-cog, a scale widely used as an efficacy measure in clinical drug trials of AD) in the treatment of AD with atorvastatin[114]. It has also been reported in the LEADe study that in posthoc analysis of the subset of individuals evaluated for *APOE* genotype, atorvastatin produced a significantly reduced decline on the ADAS-cog compared with placebo. The difference was not related to gender, nor to apoE4 allele status, and may have been due to differences in the genotyped vs. nongenotyped study populations[115].

The CLASP study, another large, multicenter, randomized, double-blind, placebo-controlled trial, was designed to investigate the effects of an 18-month simvastatin treatment (20 mg daily for 6 weeks, then 40 mg daily for the remainder of the study period) vs. placebo. A total of 406 subjects with mild-to-moderate AD (mean age of 74 years) were randomized and detailed results are awaited. Preliminary results did not indicate a benefit of simvastatin in AD treatment[113,116].

## CEREBROVASCULAR DISORDERS AND STATINS

There are a number of issues that require clarification: Does cholesterol level contribute to ischemic or hemorrhagic brain pathology? Do statins contribute to decreasing the pathology and, if so, by what mechanism? Do statins serve as primary vs. secondary prevention and what is their therapeutic role in the acute phase of stroke?

In general, the role of statins is more complicated than just lowering cholesterol. As previously discussed, pleiotrophic actions of statins include antithrombotic effects via inactivation of prothrombin and antiplatelet effects through thromboxane A2 reduction[117,118,119]. Statins also reduce blood viscosity and red blood cell aggregation[120,121].

In contrast to coronary artery disease, brain atherosclerotic disease can manifest as ischemia or hemorrhage. Strokes occur in the population on average in persons 10 years older than patients experiencing myocardial infarction. Complications of cerebrovascular disorders depend heavily on additional risk factors like hypertension, diabetes, smoking, or alcohol use[122].

Cerebral infarction is not a uniform entity. Stroke is a heterogeneous disorder with various underlying pathophysiology. Endothelial dysfunction with thickening of the endothelium of the small “end artery” of the brain is thought to play an important role in the pathogenesis of lacunar stroke[123,124]. Hypertension and diabetes mellitus are the primary risk factors for this type of stroke, which accounts for one-quarter of cerebral infarctions[125]. Atherosclerosis, responsible for myocardial infarction, stroke, and peripheral vascular disease, affects large- and medium-sized arteries. Atherosclerosis may be detected in arteries supplying the brain: the internal carotid artery, middle cerebral artery, and basilar artery[126]. Atherothrombotic stroke occurs as a result of atherosclerosis progression and presents with a frequently devastating clinical picture. Embolic stroke is usually cardiogenic in its etiology. Hemorrhagic stroke consists of intracerebral, subdural, and subarachnoid type[127].

### Role of Cholesterol in Ischemic and Hemorrhagic Stroke

Lacunar infarcts and hypertensive intracerebral hemorrhage occur in the same brain topographic location, within deep structures in the vascular distribution of the small perforating intracerebral arteries, including the putamen, thalamus, and pons. This suggests a similar pathophysiological process related to chronic hypertensive damage[128,129]. The Northern Manhattan Study (NOMAS) is a prospective, population-based incidence and case follow-up study designed to determine stroke incidence, risk factors, and outcomes in a multiethnic, urban population in a well-defined area of Manhattan. One of many questions that NOMAS attempted to answer was: Who gets lacunar infarct vs. intracranial hypertensive hemorrhage and why? Between July 1, 1993 and November 1, 1996, NOMAS identified 151 cases of lacunar infarct and 83 cases of deep intracerebral hemorrhage. Total cholesterol level data were available for 74% of the lacunar infarct cases and 84% of the deep intracerebral hemorrhage cases. The study found that a history of hypertension was prevalent in both lacunar infarct (86%) and deep intracerebral hemorrhage (77%) patients, but hypercholesterolemia occurred mostly in lacunar ischemic strokes, while hypocholesterolemia was more prevalent in cerebral hemorrhage[130]. In this study, risk factor data were collected by chart review and although total cholesterol was measured within 1 day of hospital admission, the stroke subtype itself may have influenced cholesterol level.

The increased risk of all ischemic strokes associated with higher total cholesterol as well as lack of this association has been reported in many studies[131,132]. In the Women's Health Study, a prospective cohort study of 27,937 apparently healthy women, all registered health professionals throughout the U.S. and the Commonwealth of Puerto Rico aged 45 years and above (mean age 54.7), a strong association was noted between the risk of ischemic stroke and both total cholesterol ($p < 0.001$) and LDL cholesterol levels ($p < 0.003$) over 11 years of follow-up and after adjustment for a large number of potential confounders[133]. In this large study, 94.5% of the women were white, 1.8% were black, and 3.7% were of other ethnicity. Stroke was self-reported and confirmed by review of medical records. Intraindividual variability was not accounted for since lipid levels were measured only once, at inception. Tirschwell et al. analyzed data from two ongoing case control studies of myocardial infarction and stroke. Ischemic stroke, hemorrhagic stroke, and subarachnoid hemorrhage were analyzed. The study found an association between higher total cholesterol levels and increased risk of ischemic stroke, especially lacunar and atherothrombotic subtypes, in younger patients (age below 66 years) or patients with HDL <50 mg/dl. The lowest levels of total cholesterol contributed to increased risk of the hemorrhagic stroke subtype[134].

A number of studies have documented a decreased risk of ischemic stroke in patients with higher HDL[135,136]. In a NOMAS study of the elderly (67% of cases and 69% of controls were aged 65 years or older), 539 ischemic stroke cases were matched by age, sex, and race/ethnicity to 905 controls. The ischemic stroke patients were 55% women, 53% Hispanic, 28% black, and 19% white. In this NOMAS population, the HDL cholesterol and stroke association noted in all three racial/ethnic groups persisted after adjustment for multiple potential confounders, and was found to be especially important in large-vessel atherosclerotic strokes and was seen in nonatherosclerotic infarction. In this study, no relationship was found between total cholesterol levels and stroke risk[137].

The Framingham study, a prospective study in an overwhelmingly white cohort residing in the town of Framingham, MA, found no connection between cholesterol level and stroke[138]. In the same study, high cholesterol level was associated with elevated inflammatory protein in the serum. In EUROSTROKE, a collaborative prospective case control study of European populations, total cholesterol was not associated with any significant increase in stroke risk[139]. In another study, Emond and Zareba looked at 2873 women of different ages participating in the Framingham Study, and found that in younger women (age 55 or below) with stroke, mortality was associated with hypercholesterolemia, while in an older population, the trend was reversed[140]. One may question the influence of metabolic syndrome in younger, hypercholesterolemic women with stroke. Metabolic syndrome is associated with increased mortality and incidence of ischemic stroke[141]. In the Multiple Risk Factor Intervention Trial (MR FIT), over 350,000 men were screened for multiple stroke risk factors and it was found that a serum cholesterol level under 4.14 mmol/l increased the risk of fatal intracranial hemorrhage, while a cholesterol level above 7.23 mmol/l increased the risk of death from cerebral ischemia[142]. A very interesting study from Japan[143] suggested that in a population where animal product intake is half of that in Western populations, consumption of animal fat and cholesterol resulted in reduced risk of mortality from cerebral infarction. This is an example of a population that traditionally does not enter statin studies.

The association between low total cholesterol and risk of hemorrhage is confusing. The Tirschwell study[134] showed that the subgroup of patients with the lowest level of total cholesterol had an increased risk of hemorrhagic stroke. The MR FIT study, which included exclusively men, suggested an association between low cholesterol and intracerebral hemorrhage, but not subarachnoid hemorrhage[142]. The association had significance in hypertensive, but not amyloidal, bleeds. Gender differences have been found in some studies, including a cohort of persons enrolled in the Northern California Kaiser Permanente Medical Care Program from 1978 through 1993[144]. An evaluation of 71,843 persons aged 40–89 years in this ethnically diverse population showed a statistically increased risk for intracerebral hemorrhage only in males over age 65 with low levels of serum cholesterol. However, there were very few women with low cholesterol levels in this population. In a Swedish study of 54,000 men and women, relative risk for subarachnoid hemorrhage increased with decreasing serum cholesterol level in men and for intracerebral hemorrhage in women, but not for subarachnoid hemorrhage in women[145]. The biological explanation for a possible association between low cholesterol and intracerebral hemorrhage is difficult, but low cholesterol can contribute to arterial media layer smooth muscle cell necrosis, weakening the vessel wall[142].

## Neuroprotective Role of Statins in Stroke

Another important issue to address is a neuroprotective role of statins as primary prevention. In a retrospective analysis of the NOMAS study, 650 patients age 40 and older with first ischemic stroke were interviewed regarding medication taken at home prior to the event. Patients taking lipid-lowering agents (8.8% of the patients, with 90.9% of these taking statins) at the time of stroke had a lower poststroke mortality at 90 days and a lower risk of worsening during hospitalization. However, there was no difference in outcomes at 6 months[146]. Similarly, in a large prospective study from Vienna, a significant decrease in severity of stroke-induced deficits at 1 week was observed among patients who were taking statins at the time of their stroke[147]. The effect is likely due to anti-inflammatory mechanisms as well as up-regulation of eNOS. To support this hypothesis, patients hospitalized with infections (pneumonia, urinary tract infection, and cellulitis) had decreased rates of sepsis if they were taking statins at the time of infection[148]. In patients with acute ischemic stroke in Barcelona, Spain, premorbid statin use gave no significant benefit in the short term, but was associated with better functional outcome[149].

Can statins exert a neuroprotective effect during the acute phase of a cerebral bleed? The injury during acute brain hemorrhage occurs not only through mechanical destruction due to mass effect, but mainly due to local inflammation and apoptosis[150]. Anti-inflammatory propensities of statins can improve outcome in acute bleed. Atorvastatin administration to rats with collagenase-induced deep intracerebral hemorrhage resulted in reduced edema, perihematomal cell death, decreased filtration of leukocytes and microglia, and increased eNOS expression. The functional outcome in this rodent model was improved as well[151]. Neuroprotective effects of statins were absent in eNOS-deficient mice[152], therefore suggesting that enhancement of eNOS activity by statins is one of the main neuroprotective mechanisms. In addition, statins cause a dose-dependent inhibition of leukocyte recruitment and migration into the central nervous system by suppressing adhesion molecules as well as proinflammatory mediators, such as TNF-alpha and iNOS[153]. In summary, attenuation of inflammatory response, decreasing brain edema, and improving local cerebral blood flow can positively affect outcomes after intracerebral hemorrhage.

## Statins and Brain Hemorrhage

The impact of statin use and low cholesterol (especially LDL cholesterol) levels on the incidence of primary intracranial hemorrhage is still under debate. Low cholesterol is known to be associated with increased cerebral hemorrhage. In the Stroke Prevention by Aggressive Reduction in Cholesterol Levels (SPARCL) trial in which high-dose atorvastatin for secondary stroke prevention was analyzed, a significant increase of brain hemorrhages in the treatment group was observed, whereas total and LDL cholesterol levels did not affect the risk of hemorrhagic stroke[154]. Interestingly, some of the randomized trials of cholesterol lowering with statins have not shown increased risk of brain hemorrhage. In a large meta-analysis with more than 90,000 patients treated with statins for primary and secondary prevention of stroke, the frequency of brain hemorrhage was not increased[155]. The National Acute

Stroke Israeli Surveys (NASIS) looked at outcome in 312 intracerebral hemorrhage subjects with mean age of 77.4 ± 9.9 years, 89 of them receiving statins at the time of their event (hemorrhagic transformation of ischemic strokes was excluded), and found that statin-treated patients had lower neurological disability at onset, lower death and dependency rates, and increased chances for a positive outcome[156]. In a pooled analysis of three large, placebo-controlled, randomized trials, pravastatin was found to reduce total stroke incidence across a wide range of patient characteristics and manifested as a 24% reduction in nonfatal nonhemorrhagic strokes, but with no evidence that pravastatin had an effect on hemorrhagic strokes. It is important to realize that patients with baseline normal cholesterol level might not have been included in the study[157]. Low levels of LDL cholesterol, and possibly total cholesterol, have been associated with greater risk of hemorrhagic transformation after acute ischemic stroke attributable to large artery atherothrombosis, but not in cardioembolism[158].

The interaction between apoE and statin use was looked at by Woo and colleagues[159]. They found that treatment with statins did not increase risk for intracerebral hemorrhage. History of hypercholesterolemia was associated with decreased risk of intracerebral hemorrhage. Use of statins was statistically the same in the hypercholesterolemia group with intracerebral hemorrhage as in controls (hypercholesterolemia without intracerebral hemorrhage). There was no different effect of statins on lobar vs. nonlobar bleed. Risk of intracerebral hemorrhage was significantly lower in the hypercholesterolemic group with statin use and apoE3 genotype.

Some studies suggested that the apoE genotype can determine responsiveness to statins. Patients with the apoE2 genotype may lower their cholesterol more effectively with statins than patients with apoE4[160].

Feigin et al.[161] found that hypercholesterolemia was associated with a reduced risk of subarachnoid hemorrhage. On the other hand, use of statins reduces risk of cerebral vasospasm. In a systematic review of nine longitudinal studies and 11 case control studies, Teunissen and colleagues found no effect of hypercholesterolemia on risk of subarachnoid hemorrhage in either males or females[162].

Hemorrhagic transformation of ischemic stroke occurs in 6.8% of patients treated with the thrombolytic agent intravenous tissue plasminogen activator (tPA), 10–12% of patients treated with intrarterial tPA, and 7–8% of patients treated with MERCI (mechanical embolus removal in cerebral ischemia) clot retrieval. Mild hemorrhagic transformation is clinically silent, but large ones can affect outcome. Although statins are recommended in ischemic stroke, patients with lower LDL cholesterol had more hemorrhagic transformation regardless of statin used or type of intervention[163].

The SPARCL study showed that use of high-dose atorvastatin (80 mg/day) increased the relative risk of hemorrhage by 66%[154]. Does low cholesterol trigger intracerebral hemorrhage or is it an epiphenomenon? Studies of statin use in coronary artery disease have not demonstrated an increase in intracerebral hemorrhages, but the population was 10 years younger than the typical stroke population. It is possible that other effects of statins, such as reduction of thromboxane A2 and thrombin formation, as well as reduced blood viscosity, could be contributing factors.

Multiple epidemiological studies examining statins and stroke are described herein. These studies involve numerous different types of cohorts with respect to gender, ethnicity, socioeconomic status, age, and geographic location. Thus, data from these studies could be confounded by demographics and cultural and lifestyle factors that are particular to each cohort, but are not addressed in the study. The specific populations are described briefly here, but this is an overview. For more detail and to assess study strength, it is advisable to consult the actual source material cited in the reference list.

## CONCLUSIONS

In summary, exploration of the use of statins in the treatment of stroke and AD is rapidly evolving. There are a lot of data to support the use of these agents in those with prior cerebrovascular disease and as pretreatment for ischemic stroke[164]. The use of statins in the acute phase of stroke for its brain-protective effect is under study, along with many other potential approaches[165]. In patients who were taking a statin at the time of acute ischemic stroke, withdrawal of the statin is not advisable and may worsen outcome[166].

Although observational studies have been promising, the use of statins for primary prevention of AD is not supported by clinical findings at this time[110].

## ACKNOWLEDGMENTS

This work was supported by a grant from the Neuroscience Education and Research Foundation and an Innovative Research Grant from the Arthritis Foundation (Reiss). We would like to thank Dr. Amy Glass for composing Fig. 1.
